# Phase II study of weekly irinotecan and capecitabine treatment in metastatic colorectal cancer patients

**DOI:** 10.1186/1471-2407-14-986

**Published:** 2014-12-19

**Authors:** Wenhua Li, Jianming Xu, Lin Shen, Tianshu Liu, Weijian Guo, Wen Zhang, Zhiyu Chen, Xiaodong Zhu, Jin Li

**Affiliations:** Department of Medical Oncology, Fudan University Shanghai Cancer Center, 270 Dong-An Road, Shanghai, 200032 China; Department of Oncology, Shanghai Medical College, Fudan University, Shanghai, China; Department of Gastrointestinal Oncology, 307 Hospital of PLA, Academy of Military Medical Science, Beijing, China; Department of Gastrointestinal Oncology, Peking University Cancer Hospital & Institute, Beijing, China; Department of Medical Oncology, Zhongshan Hospital, Fudan University, Shanghai, China

**Keywords:** Colorectal neoplasms, Irinotecan, Capecitabine, Antineoplastic agents

## Abstract

**Background:**

The purpose of this phase II study was to evaluate the safety and efficacy of weekly irinotecan and capecitabine (wXELIRI) treatment in patients with metastatic colorectal cancer, specifically the rate of severe diarrhea.

**Methods:**

Patients with unresectable histologically confirmed metastatic colorectal cancer with measurable disease received weekly irinotecan 90 mg/m^2^ on day 1 and capecitabine 1200 mg/m^2^ twice daily on days 1–5. Patients naïve to systemic chemotherapy for metastatic disease or who had failed FOLFOX (infusional 5-fluorouracil [5-FU], leucovorin, and oxaliplatin) or XELOX (capecitabine plus oxaliplatin) as first-line treatment were eligible. The primary endpoint was the rate of grade 3/4 diarrhea. Secondary endpoints included progression-free survival (PFS), overall survival (OS), and safety.

**Results:**

A total of 52 patients were enrolled, 30 of whom received wXELIRI as first-line treatment and 22 as second-line treatment. Grade 4 diarrhea was observed in one patient and the rate of grade 3/4 diarrhea was 7.7%. The other common grade 3/4 toxicities included leukopenia (9.6%), neutropenia (17.3%), nausea (3.8%), vomiting (3.8%), fatigue (1.9%), and hand-foot syndrome (1.9%). The median progression-free survival and overall survival for the 30 patients treated in the first-line setting was 8.5 and 16.3 months, while those for the 22 patients treated in the second-line setting was 5.0 and 10.7 months, respectively.

**Conclusions:**

The wXELIRI regimen resulted in a low rate of severe diarrhea with an acceptable toxicity profile. This study provides a basis for a subsequent randomized controlled study of wXELIRI versus FOLFIRI (irinotecan, 5-FU, and folinic acid) to further explore the efficacy and safety of this regimen.

**Trial registration:**

ClinicalTrials.gov: NCT01322152.

## Background

Colorectal cancer, the second leading cause of cancer death in the USA, has increased in frequency in China in recent years. Based on a registry in Shanghai, a city with a population of 23 million, colorectal cancer has become the third most prevalent malignancy [1]. Approximately 50-60% of patients diagnosed with colorectal cancer will develop metastases [2, 3], for whom systematic chemotherapy is the standard treatment.

Irinotecan combined with continuous infusion of 5-fluorouracil (5-FU) and folinic acid (the FOLFIRI regimen) is the established option for first- and/or second-line treatment of metastatic colorectal cancer. Capecitabine, an oral fluoropyrimidine that mimics continuous 5-FU infusion by generating 5-FU preferentially in the tumor tissues [4], has been shown to have comparable efficacy to 5-FU/folinic acid as first-line treatment of metastatic colorectal cancer, with an additional benefit of convenient administration without hospitalization [5]. The combination of irinotecan and capecitabine (XELIRI) has been assessed, but the associated gastrointestinal toxicity, especially the incidence of severe diarrhea, affected the feasibility of this regimen [6, 7]. The incidence of grade 3/4 diarrhea was higher (17-36% vs 12-15%) with a 3-week XELIRI regimen than with the FOLFIRI regimen [8–11], and non-superior time to progression (TTP; 6-9 months vs 6.7-8.5 months) and overall survival (OS; 13-20 months vs 14.8-17.4 months) were observed [12–14]. Toxicity-induced dose reduction and treatment delay weakened the efficacy of the XELIRI regimen.

To reduce the side effect of diarrhea, a 2-week XELIRI regimen was tried recently. The 2-week regimen (irinotecan on day 1, capecitabine on days 2-8 or days 1-5 and 8-12) exhibited promising activity (TTP, 8-10 months, OS, 15-19 months) with improved tolerability (grade 3/4 diarrhea, 8.1-15.0%) [15, 16], but an increased rate of severe diarrhea was noted in elderly patients, which resulted in dose reduction [15].

This study aimed to evaluate the tolerability of a weekly XELIRI regimen as first- or second-line treatment in patients with metastatic colorectal cancer. The dose and schedule was chosen based on the assumption that 5 days on/2 days off administration of capecitabine may better mimic continuous 5-FU infusion [16]. The dose of irinotecan was calculated as a weekly dose according to the FOLFIRI regimen [14]. The study investigated the possibility of a further reduction of the rate of severe diarrhea with weekly XELIRI treatment, and evaluated the safety and efficacy of this schedule in Chinese patients with metastatic colorectal cancer.

## Methods

### Patients

Patients aged 18-70 years with histologically or cytologically confirmed advanced colorectal adenocarcinoma, with an Eastern Cooperative Oncology Group performance status of 0 to 1 and life expectancy of ≥ 3 months were eligible. Patients who were chemotherapy-naive or who had failed first-line treatment with either XELOX or FOLFOX were enrolled if they had at least one measurable disease lesion according to the Response Evaluation Criteria in Solid Tumors (RECIST) criteria, version 1.1. Previous (neo) adjuvant chemotherapy was permitted if it had been completed ≥ 6 months before enrollment, although prior irinotecan therapy was not allowed. Patients were required to have adequate bone marrow, hepatic, and renal function. Patients with previous chronic inflammatory bowel disease, chronic diarrhea or recurrent bowel obstruction, pelvic radiotherapy within 6 months, or symptomatic brain metastases were excluded. The study was approved by the independent ethics committee of Fudan University Shanghai Cancer Center, Shanghai, China and registered at ClinicalTrials.gov (NCT01322152). The study was carried out in accordance with the Declaration of Helsinki. All patients provided written informed consent before study entry.

### Treatment

All enrolled patients received a weekly regimen of irinotecan and capecitabine (wXELIRI), as follows: irinotecan (Campto®, Pfizer) 90 mg/m^2^ given intravenously on day 1; capecitabine (Xeloda®, Roche) 1200 mg/m^2^ given orally twice daily on days 1-5. The treatment cycles were administered every week until disease progression or unacceptable toxicity, or consent withdrawal. For patients with poor tolerance to toxicities, treatment delay was permitted for no more than 2 weeks.

Unless contraindicated, atropine could be given to prevent the cholinergic adverse effects (including early diarrhea). Loperamide was recommended as the standard anti-diarrhea treatment, and other symptomatic treatment could be given according to the institution’s practice guidelines.

### Dose modification

Dose adjustments were made based on the worst grade of toxicity encountered during the previous cycle. For hematological toxicities, the dose of chemotherapeutic drugs was reduced in the following cases: grade 4 neutropenia or leukopenia; grade 3 or greater febrile neutropenia; grade 3 or greater thrombocytopenia. For nonhematological toxicity, the dose of related drug was reduced when grade 3 or greater toxicities occurred (except for alopecia). The dose of irinotecan or capecitabine was reduced by 25% of the starting dose. If a patient required more than two successive dose reductions, therapy was dicontinued.

Treatment was delayed until the absolute neutrophil count was ≥ 1.5 × 10^9^/L and platelet count was ≥ 80 × 10^9^/L, and recovery to grade ≤ 1 for mucositis, diarrhea and other toxicities (with exception of alopecia).The maximum authorized delay is of 2 weeks.

### Assessments

Pretreatment assessment included a detailed medical history, physical examination, routine laboratory tests, and performance status. Laboratory evaluation included a routine blood count, urinalysis, and electrolyte, renal, and liver function tests. Adverse events and concomitant medications were recorded at the end of each cycle. Toxicity was evaluated and graded according to the National Cancer Institute Common Terminology Criteria for Adverse Events, version 4.0.

Radiographic scans (computed tomography or magnetic resonance imaging) for efficacy evaluation were conducted at baseline and every 2 months thereafter according to the RECIST 1.1 guidelines. The best overall response was reported. Survival status was assessed every 3 months after discontinuation of study treatment.

### Statistical analysis

This phase II study was designed to assess the rate of severe diarrhea (calculated as the percentage of patients with grade 3 and grade 4 diarrhea) with the wXELIRI regimen. The rate of severe diarrhea with the 2-week XELIRI or the FOLFIRI regimens was reported as 15% [15, 17], and we supposed that the rate with the wXELIRI regimen was, at most, 5%. A one-stage Fleming design, with an exact significance level of p = 0.05 and a power of 80%, was used to test the hypothesis. With a sample size of 52 evaluable patients, the regimen would be declared promising if less than 6 patients with severe diarrhea were observed.

The efficacy analysis included all patients who received at least one dose of study medication and had at least one efficacy evaluation after baseline. For the safety analysis, all patients who received one dose of study medication were included. The primary study end point was the rate of severe diarrhea. Secondary end points were PFS (defined as the time between the first dose of study medication and first documented disease progression or death), ORR, DCR (defined as the percentage of patients with CR, PR, and SD for at least 8 weeks), OS (defined as the time between the first dose of study medication and death), and safety.

Survival function of time-to-event end points was estimated by using the Kaplan-Meier method. Chi-square test was performed for enumeration data on response rate and clinical benefit rate.

## Results

### Patients

From March 2011 to October 2012, a total of 52 patients (25 men and 27 women), aged from 26 to 70 years (median, 60 years) with advanced colorectal cancer received wXELIRI treatment. Previous chemotherapy regimens for the patients receiving second-line treatment included FOLFOX (infusional 5-FU, leucovorin, and oxaliplatin) and XELOX (capecitabine plus oxaliplatin). Table 1 shows the patients’ demographic and clinical characteristics at enrollment.

**Table 1:** **Clinical and demographic characteristics at enrollment (N = 52)**

Characteristic	All patients	First-line treatment	Second-line treatment
	Number (%)	Number (%)	Number (%)
		30 (57.7)	22 (42.3)
Median age (range), years	60 (32-70)	60 (32-70)	60 (26-68)
ECOG score			
0	20 (38.5)	10 (33.3)	10 (45.5)
1	32 (61.5)	20 (67.7)	12 (54.5)
Sex			
Male	32 (61.5)	18 (60.0)	14 (63.6)
Female	20 (38.5)	12 (40.0)	8 (36.4)
Primary lesion site			
Colon	32 (61.5)	16 (53.3)	16 (72.7)
Rectum	20 (38.5)	14 (46.7)	6 (27.3)
Positive history of diabetes	9 (17.3)	5 (16.7)	4 (18.2)
Primary tumor resection	46 (88.5)	26 (86.7)	20 (90.9)
Site of metastasis			
Liver	31 (59.6)	16 (53.3)	15 (68.2)
Lung	18 (34.6)	13 (43.3)	5 (22.7)
Abdominal cavity	18 (34.6)	8 (26.7)	10 (45.5)
Number of metastatic lesions			
Single	24 (46.2)	15 (50.0)	9 (40.9)
Multiple	28 (53.8)	15 (50.0)	13 (59.1)

ECOG: Eastern Cooperative Oncology Group.

### Treatment

Up to 31 April 2013, the 52 enrolled patients had received a total of 644 cycles of wXELIRI. The median number of treatment cycles was 12 (range, 1-50). Median dose intensities (i.e. the actual dose administered divided by the planned dose) were 80.0% for irinotecan, and 78.9% for capecitabine. Thirty treatment cycles (4.7%) were delayed for 23 patients — 22 cycles for 15 patients receiving wXELIRI as first-line treatment and 8 cycles for 8 patients receiving wXELIRI as second-line treatment. The drug dose was reduced in 80 cycles (12.4%) in 7 patients — 28 cycles for 4 patients receiving wXELIRI as first-line treatment and 52 cycles for 3 patients receiving wXELIRI as second-line treatment. Leucopenia, diarrhea, nausea, vomiting, and hand-foot syndrome were the main causes of dose delay and reduction.

### Safety

The overall incidence of grade 3/4 adverse events was 55.5%. The most common adverse events (≥20%) were neutropenia, leucopenia, diarrhea, nausea, vomiting, anorexia, and fatigue (Table 2). The rate of severe diarrhea, the primary study end point, was 7.7% (6.7% and 9.0% for the first- and second-line settings, respectively), with one case of grade 4 diarrhea in the second-line setting (Table 3). Grade 3 diarrhea occurred in three patients, one each during the first, third, and fifth treatment cycles, and was relieved by symptomatic treatment for diarrhea and dehydration. Subsequent re-treatment did not result in diarrhea greater than grade 2. The incidence of grade 1/2 diarrhea was 40.4%, and the symptoms were relieved after standard use of loperamide and symptomatic supportive treatment in most patients.

**Table 2:** **Incidence of adverse reactions caused by weekly irinotecan and capecitabine (N = 52)**

Adverse reaction	Grade I	Grade II	Grade III	Grade IV
	Number (%)	Number (%)	Number (%)	Number (%)
Hematologic toxicity				
Leukopenia	12 (23.1)	14 (26.9)	5 (9.6)	0 (0)
Neutropenia	9 (17.3)	10 (19.2)	6 (11.5)	3 (5.8)
Febrile neutropenia	3 (5.8)	0 (0)	1 (1.9)	0 (0)
Thrombocytopenia	5 (9.6)	3 (5.8)	0 (0)	0 (0)
Anemia	7 (13.5)	13 (25)	1 (1.9)	0 (0)
Non-hematologic toxicity				
Diarrhea	16 (30.8)	5 (9.6)	3 (5.8)	1 (1.9)
Nausea	13 (25.0)	7 (13.5)	2 (3.8)	0 (0)
Vomiting	7 (13.5)	11 (21.2)	2 (3.8)	0 (0)
Anorexia	11 (21.2)	3 (5.8)	0 (0)	0 (0)
Fatigue	11 (21.2)	8 (15.4)	1 (1.9)	0 (0)
Alopecia	5 (9.6)	0 (0)	0 (0)	0 (0)
Hand-foot syndrome	3 (5.8)	0 (0)	1 (1.9)	0 (0)
Oral ulceration	5 (9.6)	1 (0)	1 (1.9)	0 (0)
Venous thrombus	1 (1.9)	0 (0)	0 (0)	0 (0)
Pulmonary infection	1 (1.9)	1 (1.9)	0 (0)	0 (0)
Hyperbilirubinemia	2 (3.8)	0 (0)	0 (0)	0 (0)
Constipation	1 (1.9)	1 (1.9)	0 (0)	0 (0)
Elevated creatinine	1 (1.9)	0 (0)	0 (0)	0 (0)
Elevated blood glucose	1 (1.9)	1 (1.9)	0 (0)	0 (0)
Respiratory alkalosis	1 (1.9)	0 (0)	0 (0)	0 (0)
Pain	3 (5.8)	0 (0)	0 (0)	0 (0)
Hypophosphatemia	0 (0)	0 (0)	1 (1.9)	0 (0)
Hypomagnesemia	0 (0)	1 (1.9)	0 (0)	0 (0)
Hypokalemia	0 (0)	0 (0)	1 (1.9)	0 (0)

**Table 3:** **Incidence of diarrhea after first- and second-line weekly irinotecan and capecitabine**

	Total	Grade I	Grade II	Grade III	Grade IV
		Number (%)	Number (%)	Number (%)	Number (%)
First-line treatment	30 (57.7)	10 (33.3)	3 (10)	2 (6.7)	0 (0)
Second-line treatment	22 (42.3)	6 (27.3)	2 (9.1)	1 (4.5)	1 (4.5)

Nausea and vomiting was another frequent gastrointestinal reaction, with an incidence of 38.5-42.3%. After prophylactic administration of an antiemetic drug (5-hydroxytryptamin 3 [5-HT 3] receptor antagonists), nausea and vomiting was tolerable in most cases. One patient had dose reduction and delayed treatment due to intolerable grade 3 vomiting.

The major hematological toxicities were leucopenia and neutropenia. Three patients developed grade 4 neutropenia and one developed grade 3 febrile neutropenia. After symptomatic therapy and prophylactic antibiotic treatment, all patients recovered. The incidence of mild to moderate anemia was 38.5%. One patient discontinued treatment because of grade 3 anemia complicated by grade 3 fatigue after 5 cycles of wXELIRI, and one patient had the capecitabine dose reduced due to the grade 3 hand-foot syndrome after 16 cycles. Table 4 shows the adverse reactions.

**Table 4:** **Analysis of efficacy of weekly irinotecan and capecitabine as first- or second line treatment**

Variable	First-line treatment (n = 30)		Second-line treatment (n = 22)	
	Number (%)	95% CI	Number (%)	95% CI
Overall survival				
Deaths	16 (53.3)		12 (54.5)	
Median OS (months)	16.3	10.47–22.13	10.7	5.80–15.60
Progression-free survival				
Progression events	25 (83.3)		20 (90.9)	
Median PFS (months)	8.5	6.22–10.78	5.0	1.74–8.26
Response	27		21	
ORR	10 (37.0)		3 (14.3)	
PR	10 (37.0)		3 (14.3)	
SD	11 (40.7)		13 (61.9)	
PD	6 (22.2)		5 (23.8)	
Treatment cycles				
Total	378		266	
Median number of cycles (range)	12 (1–50)		12 (1–33)	
Median treatment period (months)	3.7		3.7	

CR: complete response; ORR: overall response rate; OS: overall survival; PD: progressive disease; PFS: progression-free survival; PR: partial response; SD: stable disease.

### Efficacy

**Figure 1:**
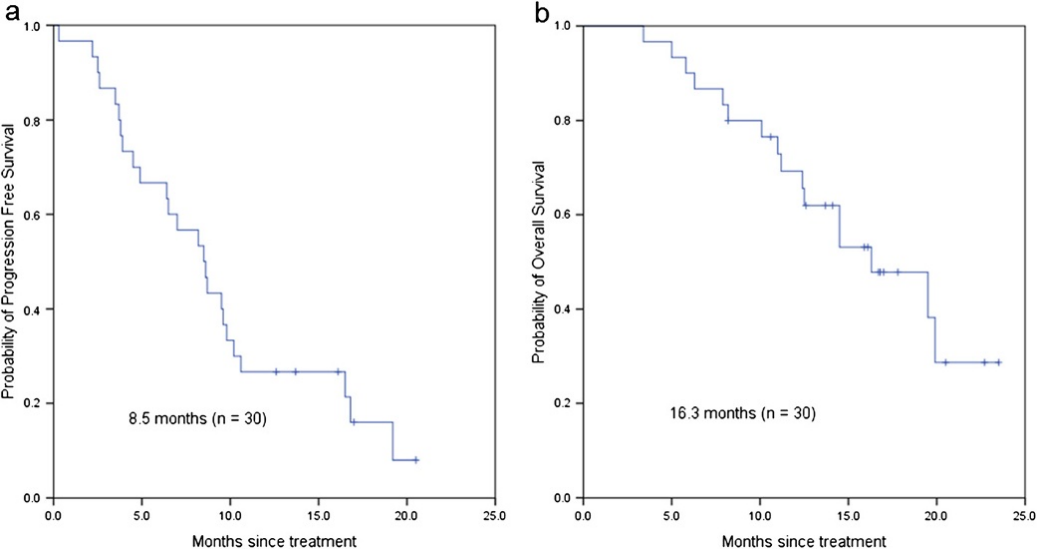
**Overall survival and progression-free survival in the first-line setting.** **(a)** progression-free survival; **(b)** overall survival.

After a median follow-up of 13.9 months (range, 1-24 months), 25 of 30 patients (83.3%) treated with wXELIRI in the first-line setting experienced disease progression, and 16 patients (53.3%) died (Table 4). The median OS was 16.3 months (95% confidence interval [CI]: 10.47-22.13 months) and the median progression-free survival (PFS) was 8.5 months (95% CI: 6.22-10.78 months) (Figure 1). Ten patients experienced partial response (PR) and 11 patients had stable disease (SD), whereas no complete response (CR) was observed and 3 patients did not have response evaluation due to withdrawal of consent after two cycles. The objective response rate (ORR) was 37% (10/27 patients) and the disease control rate (DCR) was 77.7% (21/27 patients). Secondary R0 metastasectomy was performed in 2 patients after 6 cycles.

**Figure 2:**
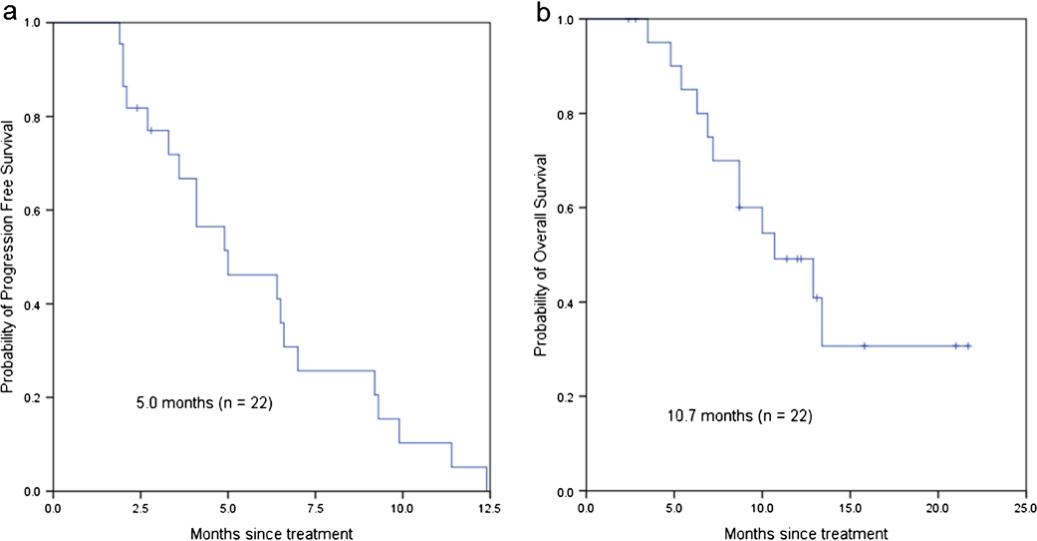
**Overall survival and progression-free survival in the second-line setting.** **(a)** progression-free survival; **(b)** overall survival.

In patients pretreated with FOLFOX or XELOX (n = 22), the median PFS was 5.0 months (95% CI: 1.74-8.26 months) and the median OS was 10.7 months (95% CI: 5.80-15.60 months), with a median follow-up period of 13.8 months (range, 1-17 months) (Figure 2). Among the 21 evaluable patients in the second-line setting, three (14.3%) achieved PR, and 13 (61.9%) had stable disease, whereas five (23.8%) had disease progression at the first efficacy evaluation. One patient withdrew from the study due to personal reasons after one cycle.

## Discussion

This study demonstrated that patients with colorectal cancer experienced a relatively low incidence of severe diarrhea with the wXELIRI regimen as first- or second-line treatment. The incidence of grade 3 and grade 4 diarrhea was 5.8% and 1.9%, respectively. Among the 30 patients who received the study treatment in the first-line setting, the rate of grade 3 diarrhea was only 6.7% and no grade 4 diarrhea was observed. This was much lower than that observed with the 3-week or 2-week XELIRI [8, 10, 11, 15] and FOLFIRI [12, 13] regimens. In the MAC-6 study [16], the 5 days on/2 days off administration of capecitabine was introduced to ensure the absence of a completely drug-free interval during treatment, while maintaining a reasonable dose intensity, and a rate of grade 3 diarrhea of 8.1% was obtained. We have also demonstrated a similar low incidence of severe diarrhea in this series when using the same dosing schedule of capecitabine and dividing the dose of irinotecan from the FOLFIRI regimen into once weekly administration. In the literature, the median time for delayed diarrhea caused by irinotecan was 6-14 days after administration [18, 19]. Diarrhea may be aggravated in the second week when using the traditional regimen of capecitabine from days 1 to 14 every 21 days, which may result in discontinuation of the oral fluoropyrimidine causing under-dosage. Our results indicate that weekly use of capecitabine is feasible and tolerable with less drug interruption when combined with irinotecan.

We analyzed the efficacy and survival data according to the treatment setting. Survival in this study was similar to that in previous studies in patients undergoing first-line treatment with irinotecan and capecitabine (Table 5). About 10% of the patients in our study accepted targeted therapy as subsequent treatment. The median OS for first-line treatment was comparable with the published data for patients undergoing chemotherapy [20–23]. In the second-line setting, wXELIRI showed promising efficacy. Among 22 patients who had failed prior oxaliplatin, the ORR was 14.3% and the median PFS was 5.0 months, which were significantly superior to the ORR of 4% and PFS of 2.5 months with FOLFIRI after failure of FOLFOX in the GERGOR study [17], and the median OS exceeded 10 months. Given these data from a limited number of patients, it would be interesting to ascertain the survival data for second-line treatment of colorectal cancer in a greater number of patients.

**Table 5:** **Summary of clinical trials of capecitabine and irinotecan in the first-line setting**

Study	Year	Treatment	Number of patients	ORR (%)	PFS (months)	OS (months)	Grade 3/4 diarrhea (%)
Bajetta, et al [9]	2004	CAPIRI vs XELIRI	140	CAPIRI 44	CAPIRI 7.6	—	CAPIRI 17
				XELIRI 47	XELIRI 8.3		XELIRI 36
Borner, et al [8]	2005	CAPIRI vs XELIRI	75	CAPIRI 34	CAPIRI 6.9	CAPIRI 17.4	CAPIRI 34
				XELIRI 35	XELIRI 9.2	XELIRI 24.7	XELIRI 19
Cartwright, et al [10]	2005	XELIRI	49	45	6.2	13.4	20
Patt, et al [11]	2007	XELIRI	52	46	TTP 7.1	15.6	20
Rea, et al [24]	2005	XELIRI	57	42	TTP 8.3	—	9
Garcia-Alfonso, et al [15]	2009	XELIRI (every 2 weeks)	53	40	TTP 8.4	19.3	15.9
Choi, et al [16]	2008	XELIRI (every 2 weeks)	43	51	TTP 10.1	15.4	8.1

CAPIRI: capecitabine and irinotecan; ORR: objective response rate; OS: overall survival; PFS: progression-free survival; TTP: time to progression; XELIRI: irinotecan and capecitabine.

The low rate of severe diarrhea enabled a greater intensity of chemotherapy to be achieved, thus ensuring improved efficacy; a treatment delay of only 4.7% was observed in this study. Patients experienced more grade 3/4 neutrophilia than in the MAC-6 study [16], but hematological suppression was easily controlled with granulocyte colony-stimulating factor support.

It should be admitted that the doses of the weekly regimen of irinotecan and capecitabine in our study were not established through a formal phase I study. However, the tolerable dose for weely-used irinotecan was evaluated in the previous phase I trial [25], of which irinotecan with a dose of 100 mg/m^2^ was given on days 1 and 8, and capecitabine with 2000 mg/m^2^ on days 1-14 of a 3-week cycle was recommended. In our study, the dose of irinotecan was chosed based on that in the FOLFIRI regimen with 180 mg/m^2^ for 2 weeks [17], equal to 90 mg/m^2^ weekly. Although the planned dose intensities of irinotecan and capecitabine with wXELIRI were higher than those with the biweekly [15] or every three weeks [10, 11] irinotecan/capecitabine regimen, the rate of diarrhea decreased while the survival data was similar, suggesting the feasibility of weekly used regimen.

Additional use of targeted therapy based on irinotecan and capecitabine is a new direction [20, 26–28]. Recent study data indicated that the 3-week XELIRI regimen combined with bevacizumab had comparable efficacy to FOLFIRI combined with bevacizumab, with incidences of grade 3/4 diarrhea and agranulocytosis of 19% and 13%, respectively [20]. The XELIRI plus cetuximab regimen also achieved good efficacy with an OS of more than 20 months [28], but poor tolerance was a concern as 32% of patients required dose reduction [29]. Whether the wXELIRI regimen could become an alternative to combination therapy with targeted drugs needs further investigation.

## Conclusions

The weekly irinotecan and capecitabine combination is associated with a low incidence of severe diarrhea and has an acceptable toxicity profile. wXELIRI could be an alternative regimen for patients with metastatic colorectal cancer, especially in the second-line setting. Further randomized controlled studies are needed to evaluate the efficacy and safety of this regimen in a larger sample size.

## Authors’ information

JL: MD, PhD, Professor, Director of Department of Medical Oncology, Fudan University Shanghai Cancer Center.

## Abbreviations

CR: Complete response
DCR: Disease control rate
ECOG: Eastern cooperative oncology group
FOLFIRI: Irinotecan, continuous infusion of 5-fluorouracil, and folinic acid
FOLFOX: 5-fluorouracil, leucovorin, and oxaliplatin
5-FU: 5-fluorouracil
ORR: Overall response rate
OS: Overall survival
PFS: Progression-free survival
PR: Partial response
RECIST: Response evaluation criteria in solid tumors
SD: Stable disease
TTP: Time to progression
XELIRI: Irinotecan and capecitabine
XELOX: Capecitabine and oxaliplatin.
